# Influenza A H1N1 Community-Acquired Pneumonia: Characteristics and Risk Factors—A Case-Control Study

**DOI:** 10.1155/2019/4301039

**Published:** 2019-03-17

**Authors:** Romina Abelleira, Alberto Ruano-Ravina, Adriana Lama, Gema Barbeito, María E. Toubes, Cristina Domínguez-Antelo, Francisco J. González-Barcala, Nuria Rodríguez-Núñez, Pedro J. Marcos, María L. Pérez del Molino, Luis Valdés

**Affiliations:** ^1^Service of Pneumology, University Clinical Hospital of Santiago de Compostela, Santiago de Compostela, Spain; ^2^Department of Preventive Medicine and Public Health, University of Santiago de Compostela, Santiago de Compostela, Spain; ^3^CIBER de Epidemiología y Salud Pública (CIBERESP), Madrid, Spain; ^4^Group of Epidemiology, Public Health and Evaluation of Health Services, Health Research Institute of Santiago de Compostela (IDIS), Santiago de Compostela, Spain; ^5^Service of Microbiology, University Clinical Hospital of Santiago de Compostela, Santiago de Compostela, Spain; ^6^Critical Care Department, University Clinical Hospital of Santiago de Compostela, Santiago de Compostela, Spain; ^7^Interdisciplinary Research Group in Pneumology, Health Research Institute of Santiago de Compostela (IDIS), Santiago de Compostela, Spain; ^8^Service of Pneumology, University Hospital of A Coruña, A Coruña, Spain

## Abstract

**Introduction:**

Influenza A H1N1 community-acquired pneumonia (CAP) is a quite frequent respiratory disease. Despite being considered more serious than other CAPs, there are very few studies comparing its characteristics with noninfluenza CAP. We aim to establish the differences between pneumonia due to H1N1 virus and pneumonia not caused by H1N1 influenza virus and to determine the probability that a pneumonia is due to an H1N1 virus infection based on the most relevant variables.

**Methods:**

We used a case-control study where cases were H1N1 CAP patients with confirmed microbiological diagnosis and controls were patients with CAP admitted to hospital. H1N1 and other influenza types were discarded among controls. We calculated the probability of being a case or control using multivariate logistic regression.

**Results:**

We included 99 cases and 270 controls. Cases were younger than controls (53 vs 71 years, respectively). Mortality was much higher for H1N1 patients (13% vs 0.3%), and admission to intensive care unit was more frequent for H1N1 cases. The variables most associated with presenting H1N1 CAP were bilateral affectation on chest X-rays (OR: 5.70; 95% CI 2.69–10.40), followed by presence of arthromyalgias, with cases presenting close to three times more arthromyalgias compared to controls. Low leukocytes count and high AST values were also significantly associated with H1N1 CAP. H1N1 CAPs are characterized by bilateral affectation, low leukocyte count, presence of arthromyalgias, and high AST.

**Conclusions:**

A few and easy to obtain clinical parameters might be extremely useful to distinguish H1N1 CAP from CAPs of other origin.

## 1. Introduction

In 2010, virus influenza A/H1N1 (H1N1 onwards) was declared a pandemic disease by the World Health Organization, the first with this condition in over 40 years [1]. Compared to other influenza types, H1N1 is considered a more life-threatening disease. At least 5% of patients with H1N1 infection develop community-acquired pneumonia (CAP) [2], and symptoms such as dyspnea, wheezing, vomit, and diarrhoea are associated with H1N1 CAP [2]. H1N1 pneumonia may present a rapid progression or with a severe respiratory distress syndrome and is associated with a longer hospital stay, more admissions at the intensive care unit, and higher mortality compared to bacterial CAP [3–6]. At the emergency care department, it is important to distinguish H1N1 CAP from bacterial CAP. Establishing a diagnosis as soon as possible is of paramount importance to make an impact on patients' survival and also to avoid spreading the disease. To date, there are no clear criteria to differentiate influenza A H1N1 CAP from CAP of other aetiologies (mainly bacterial ones).

The main objective of the present study is to establish the differences between pneumonia due to H1N1 virus and pneumonia not caused by H1N1 influenza virus and to create a multivariate model allowing to determine the probability that a pneumonia is due to an H1N1 virus infection at hospital admission.

## 2. Subjects and Methods

### 2.1. Design, Setting, and Definitions

We designed a case-control study performed at a Spanish third-level hospital, covering a health area comprising approximately 450,000 inhabitants. Recruitment started in 2009 and finished in 2016. To be included, a patient had to be diagnosed with CAP and also admitted to hospital, and afterwards, we defined the case or control status. All participants had to be older than 16 years. CAP definition followed the guidelines of the Infectious Diseases Society of America/American Thoracic Society [7].

A case was defined as a patient with a CAP and a confirmed microbiological diagnosis of H1N1 influenza A. Diagnosis was performed with real-time reverse-transcriptase polymerase chain reaction at the microbiology service, following recommendations of the centers for disease control [8] and without a positive culture for other microorganisms. Samples were obtained with nasopharyngeal swabs on admission. Controls were patients diagnosed with nonvirus influenza (NVI)-CAP, where influenza A infection was discarded. In order to have a closer bacterial-like CAP infection among controls, we excluded patients from the control group with concurrent influenza CAP. There is a protocol enforced at the hospital, which is applicable to all CAPs to confirm its origin. Following the protocol, all patients undergo two blood cultures, sputum culture, and urine antigens for *Streptococcus pneumoniae* and *Legionella pneumophila*, all of them performed on the first 24 hours upon admission and before antibiotic treatment. Other tests are performed occasionally including atypical pathogens serology. We did not perform routine analysis for viruses apart from influenza A, B and respiratory syncytial virus.

Antibiotic treatment was prescribed when bacterial pneumonia was diagnosed following recommendations of Spanish guidelines [9], and osetalmivir (75 mg/12 hours, 5 days) if H1N1 Influenza A was suspected. Osetalmivir was stopped in patients with a negative microbiological test for influenza A H1N1.

Cases were all those diagnosed during the study period (therefore consecutive), and controls were selected by a frequency sampling regarding cases, based on the influenza epidemic peak to avoid any bias regarding healthcare pressure or beds availability. The rationale for this frequency-based sampling was to give the same chance of hospital admission to both cases and controls with independence on the aetiology of the CAP. For the different years included in the recruitment period, a proportional percentage of cases and controls were selected to consider H1N1 CAP epidemics.

The study protocol and ethics were approved by the Santiago-Lugo Ethics Committee (year 2017/052).

### 2.2. Information Retrieval

We collected information from the electronic clinical records regarding the following variables: age at admission, gender, tobacco consumption, body mass index, month of hospitalization, vaccination (influenza and pneumococcus), Charlson index, scoring of the Pneumonia Severity Index, and CURB65 [10, 11]. We also collected extensive information on respiratory infection symptoms, admission to the intensive care unit, and length of stay. Information on biochemical parameters and cell counting was also obtained. Finally, we classified images from unilateral or bilateral appearance of pneumonia in chest X-ray. We followed up all patients using the electronical clinical records to obtain 30-day mortality upon admission (in-hospital or at home).

### 2.3. Statistical Analysis

We performed a bivariate analysis to compare the characteristics of cases and controls. To compare categorical variables, we used the chi-squared test, and if the variables were continuous, we used the median test. Statistical significance was established at a *p* value <0.05. Afterwards, we performed a crude and multivariate logistic regression. In the crude analysis, we entered the different variables individually to assess the probability of being a case or control. We also performed a full multivariate analysis including the following variables: age (three categories), gender, pneumococcal vaccination (yes/no), influenza vaccination (yes/no), leukocytes (≤500/mm^3^, >500/mm^3^), aspartate aminotransferase (AST) (≤24 IU/L, >25 IU/L), arthromyalgias (no, yes), and radiography result (lobar, bilobar, and bilateral). PSI score and CURB-65 were not introduced in the multivariate analysis because they include age and would result in an overadjustment of results. Results were expressed as odds ratios with 95% CI, and we used SPSS v22 for the statistical analysis.

## 3. Results

**Figure 1:**
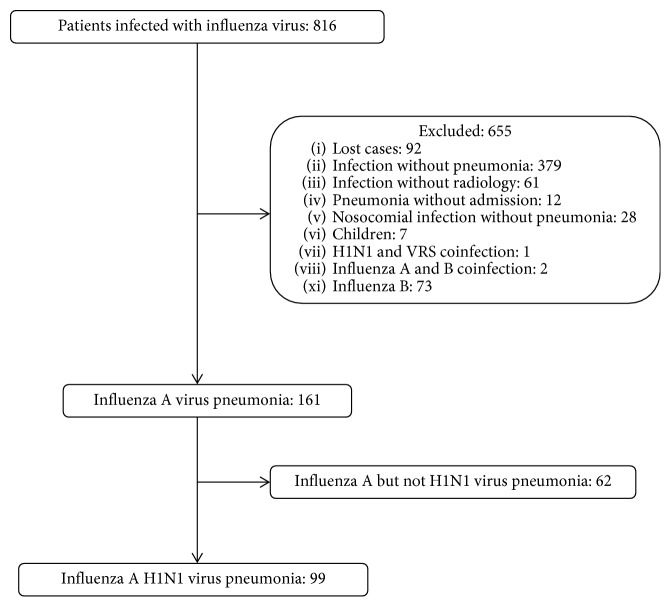
Flowchart for the inclusion/exclusion of patients in the case group.

Figure 1 shows the flowchart for the inclusion and exclusion of patients in the case group. 12 patients with H1N1 CAP were excluded because they did not want to be admitted to hospital. 62 patients with non-H1N1 influenza A also were excluded. The study included 369 patients admitted to hospital (99 H1N1 cases and 270 controls with NVI), all presenting CAP. In 111 patients of the control group (41%), a bacterial origin was microbiologically demonstrated. The most frequent infection among controls were *Streptococcus pneumoniae* (45 patients; 40.5%), *Legionella pneumophila* (11; 10%); *Klebsiella pneumoniae* (10; 9%), and methicillin-resistant *Staphylococcus aureus* (10; 9%). In the cases group, 9 patients (9%) had a bacterial coinfection. Atypical microorganisms were identified in six more patients (*Mycoplasma pneumoniae* (4 patients) and *Chlamydia pneumoniae* (2)). Sample characteristics broken down by case or control status are shown in Table 1. There were no differences on gender and trimester of admission, which shows that the matching on season of the year was appropriate. The trimester with the highest number of participants included was the second (45% of all participants), followed by the first (18.4%), and the year including most participants was 2016 followed by 2009. Cases were appreciably younger than controls (median age 53 vs 71), and the percentage of never smokers was quite similar. Controls were more frequently vaccinated for influenza and pneumococcus compared to cases, and controls had a slightly worse Charlson index. The years with the highest number of cases (and also controls) were 2016 (72 cases) and 2009 (20).

Clinical characteristics of cases compared to controls are shown in Table 2. Hospitalization at an intensive care unit was six times more frequent for cases, a rate equivalent for mechanical ventilation. Length of stay in the intensive care unit was 5 times longer for cases. When calculating PSI and CURB-65 scores, it seems that CURB-65 underestimates the severity of H1N1 patients. There were no relevant differences on PSI score between cases and controls. Cell count of white series was much lower in cases (and also for platelets) than for controls.

AST, alanine aminotransferase (ALT), and gamma-glutamyl transpeptidase (GGT) were higher in cases compared to controls. The percentage of patients with arthromyalgias was 3 times higher in cases, and bilateral presence of pneumonia in chest X-rays was 4 times more frequent for cases. Mortality was also higher in cases compared to controls. Cases presented a 13% of mortality (all in-hospital mortality) compared to only 1 death (<1%) in controls occurring in the following 30 days after admission.

We calculated the probability of being a case or control based on different variables (Table 3). Four variables showed a strong association with being diagnosed as a case, which were low count of leukocytes, high AST value, presence of arthromyalgias, and bilateral presence of pneumonia. The characteristic most strongly associated with being a case was the bilateral presence of pneumonia (OR 5.30; 95% CI 2.69–10.40), followed by arthromyalgias close to three times more frequent in cases compared to controls. The remaining two variables (high AST and low count of leukocytes) presented ORs higher than 2.

## 4. Discussion

We have applied multivariate analysis to compare clinical characteristics of CAP patients with H1N1 influenza A versus patients with nonvirus influenza CAP, and the findings are very relevant. First, it is evident that CAP is much more serious for H1N1 patients, with a higher mortality and a higher rate of intensive care unit hospitalization. Second, it should be quite easy to distinguish H1N1 CAP at admission if clinicians at emergency care consider some variables which are easily available, mainly bilateral location of pneumonia and arthromyalgias.

Several studies have described H1N1 CAP characteristics [3–6, 12–20]. Patients are usually younger with a sudden onset of symptoms [4], usually fever, cough, dyspnea, and arthromyalgias (37–46%) [3, 4, 15]. It is frequent that patients need admission at an intensive care unit requiring mechanical ventilation [4, 13], and there is a high death rate [4]. Blood analysis usually shows less than <1,000 lymphocytes/mm^3^ [3, 4], and practically all patients present high values of lactate dehydrogenase (LDH) > 350 U/L^4^. The usual radiological pattern is consolidation (with or without ground-glass opacity) located in middle or lower fields with affectation of three or more pulmonary areas [12]. Nevertheless, few studies have compared these characteristics with those of patients with NVI CAP [19–21].

The median age of H1N1 cases in the present study is slightly higher than that published in other case-series [3, 4, 13, 19, 21, 22], but significantly lower than the onset age for nonvirus influenza CAP (*p* < 0.01). Arthromyalgias are three times more frequent in cases compared to controls but this symptom is scarcely described in other case-series [19]. Vaccination rates for influenza and pneumococcus were less frequent in cases compared to controls but the rate of influenza vaccination was similar to other series (16%) [13]. We could consider that this low rate might be a risk factor to present H1N1 CAP. Nevertheless, two studies have shown that patients deceased with this infection have a significantly higher rate of vaccination compared to those surviving [15, 18].

The percentage of patients admitted to the intensive care unit (41.5%) was in the upper range of available studies (28–41%) [3, 13, 15, 19], and the same happened with patients requiring mechanical ventilation (26.4%; range 11–75%) [4, 5, 13, 15, 18, 19]. Both percentages were significantly higher than those observed for the control group (7% and 4.8%, respectively), showing the seriousness of this disease. As a consequence, the length of stay at the hospital and at the intensive care unit was significantly longer for H1N1 cases. Nevertheless, CURB-65 and PSI scores were unable to detect such seriousness. CURB-65 score seems to clearly underestimate clinical severity because 94% of patients of the case group had a score ≤2 (and 81.1% in the control group; *p* < 0.01). PSI score did not show significant differences on severity between cases and controls (64.7% vs 52.2% classified as I-III class, respectively). Previous studies have found similar results, showing that perhaps those scores are not useful to detect severity of H1N1 CAP infections [3, 14, 19, 23, 24].

The total count of leukocytes and the percentage of neutrophils were significantly lower for H1N1 CAP, and this can reflect a mainly bacterial aetiology for the control group compared to viral aetiology for cases [21]. Median number of lymphocytes (700/mm^3^) was aligned with published information [3, 4, 25] and was significantly lower compared to NVI CAP (810/mm^3^; *p* < 0.01).

Bilateral affectation through chest X-rays was four times more frequent in cases compared to controls (61.6 vs 14.8%, respectively; *p* < 0.01), with less predominance of alveolar pattern (76.8% vs 87%; *p* < 0.01) but with higher interstitial (8.7% vs 0.7%) and patchy appearance (5% vs 1.1%). These results are similar to previous studies where 71% of patients with H1N1 CAP (40/56) [12] and 40.4% (19/47) present bilateral affectation [21] and the alveolar pattern does not surpass 50% [12, 13, 26]. These findings might be extremely helpful when differentiating H1N1 CAP versus NVI CAP.

H1N1 CAP mortality was 13%, significantly higher than that observed for the control group (0.3%; *p* < 0.01) and higher than that expected for pneumonias associated with seasonal influenza (4.4%) [15] or for severe CAP (5%) [27]. A high mortality rate is observed in most case-series, with a range between 7.8% and 18.7% [5, 14, 15, 21, 28], with some exception [19]. Low mortality for CAP has been associated with factors such as age lower than 65, lower score on risk scores, and lack of some factors associated with higher mortality. These factors are being male, thoracic pain, hypothermia, systolic hypotension, tachypnea, diabetes, neurologic disease, cancer, leukopenia, and multilobar infiltration [4, 13, 15, 29]. We have observed that the presence of these factors in our sample is low (data not shown), and therefore, this fact might explain the low mortality observed among controls. Furthermore, to avoid selection bias, we selected controls at the same time as cases were recruited to keep the same probability of hospital or even ICU admissions for both cases and controls. Having died at hospital was not a cause for excluding cases or controls.

In a study performed to know the risk factors of H1N1 CAP and their impact on mortality, Reyes et al. obtained results very similar to ours. Age <60, bilateral infiltrates in thorax radiography, leucopenia, and low-values of C-reactive protein (CRP) were predictive of H1N1 CAP [21]. In our study, CRP was only determined in 43 cases and 144 controls, with a median value of 5.7 and 10.7 mg/dL, respectively (*p* < 0.01).

Differences in vaccination rates are not valuable in this study since vaccinations in Spain is directed at population older than 60 or 65, so that these data should only be compared for age-matched patients to disclose what the effect of vaccination could be.

The present research has some advantages. Data are recorded in electronic clinical records, and H1N1 influenza A CAP has been confirmed in all cases. We have also collected a high number of covariates, some of them for the first time regarding H1N1 influenza A. On the other hand, having included all consecutive cases of H1N1 CAP influenza between 2009 and 2016 guarantees the lack of selection bias. We were able to confirm bacterial origin in 41% of patients from the control group but this percentage is probably higher because it is not always possible to identify the bacterial origin in all pneumonias [7] and because patients with other influenza types (with a positive identification) were excluded. Finally, the multivariate logistic regression included 8 variables, allowing statistical adjustment for many potential confounders.

This research has some limitations. It is a retrospective study, and the sample size is not very high, particularly the number of cases. Nevertheless, comparing the number of cases included with other published studies on H1N1 CAP, the present study has one of the highest number of influenza A H1N1 confirmed cases. A further limitation is that, for some variables such as LDH and CRP, we did not have information from all participants to be included in the multivariate analysis.

To conclude, H1N1 CAP presents a specific pattern which might facilitate an a priori easy identification at the emergency care department. This pattern is characterized by bilateral affectation (in chest X-ray image), low leukocyte count, presence of arthromyalgias, and high AST. Commonly used risk scores to predict community-acquired pneumonia severities such as CURB-65 or PSI seem to underestimate H1N1 CAP severity.

## Figures and Tables

**Table 1:** Comparison of sociodemographic characteristics of cases and controls.

Variable	H1N1 cases (*n*, %)	Controls (*n*, %)	*p* value
*Gender*			0.18
Men	52 (52.5)	163 (60.4)	
Women	47 (47.5)	107 (39.6)	
*Age (years)*			<0.01
Median	53	71	
Percentile 25–75	44–69	57–82	
*Tobacco use* ^*∗*^			<0.01
Never smoker	43 (43.4)	116 (47.5)	
Former smoker	22 (22.2)	80 (32.8)	
Smoker	22 (22.2)	48 (19.7)	
Unknown	12 (12.2)	0 (0)	
*Influenza vaccine* ^*∗∗*^			<0.01
No	80 (80.8)	156 (60.7)	
Yes	18 (18.2)	101 (39.3)	
*Pneumococcal vaccine*			<0.01
No	92 (92.3)	213 (83.2)	
Yes	7 (7.7)	43 (16.8)	
*Charlson*			0.03
Median	0	1	
Percentile 25–75	0–2	0–2	
*CURB65*			<0.01
0	27 (27.3)	43 (15.9)	
1	40 (40.4)	61 (22.6)	
2	26 (26.3)	115 (42.6)	
3	5 (5.1)	46 (17.0)	
4	0 (0)	5 (1.9)	
5	1 (1.0)	0 (0)	
*PSI score*			0.08
1	18 (18.2)	29 (10.7)	
2	21 (21.2)	38 (14.1)	
3	25 (25.3)	74 (27.4)	
4	26 (26.2)	99 (36.7)	
5	9 (9.1)	30 (11.1)	

^*∗*^26 controls had missing information on tobacco use. ^*∗∗*^13 controls and 1 case had missing information on influenza vaccine.

**Table 2:** Factors related with hospitalization and mortality.

Variable	H1N1 cases (*n*, %)	Controls (*n*, %)	*p* value
*Admission to the intensive care unit*			<0.01
No	58 (58.5)	251 (93.0)	
Yes	41 (41.5)	19 (7.0)	
*Length of stay at intensive care unit* (days)^*∗*^			<0.01
Median	14	3	
Percentile 25–75	8–24.5	2–7	
*Needed invasive ventilation*			<0.01
No	63 (63.6)	256 (95.2)	
Yes	36 (26.4)	13 (4.8)	
*Length of hospital stay (days)*			<0.01
Median	10	7	
Percentile 25–75	7–19	5–9	
*In-hospital mortality*	13 (13)	0 (0)	<0.01
*Mortality following hospital discharge (<30 days)*	0 (0)	1 (0.3)	<0.01
*Leukocytes (cell/mm* ^*3*^)			<0.01
Median	6,560	12,250	
Percentile 25–75	4,230–9,540	8,077–18,577	
*Neutrophils (cell/mm* ^*3*^)			<0.01
Median	5,300	9,900	
Percentile 25–75	6,307.5–15,685	2,910–7,980	
*Lymphocytes (cell/mm* ^*3*^)			<0.01
Median	700	810	
Percentile 25–75	430–1,200	600–1,267.5	
*Monocytes (cell/mm* ^*3*^)			<0.01
Median	300	590	
Percentile 25–75	110–540	380–800	
*AST (IU/L)*			<0.01
Median	45	21	
Percentile 25–75	24.5–103.5	15–37	
*Arthromyalgia*			<0.01
No	62 (62.6)	235 (87.3)	
Yes	37 (37.4)	34 (12.7)	
*Chest X-ray presentation*			<0.01
Lobar	37 (37.4)	205 (75.9)	
Bilobar	1 (1.0)	25 (9.3)	
Bilateral	61 (61.6)	40 (14.8)	
*Radiological pattern*			<0.01
Alveolar	76 (76.8)	235 (87.0)	
Interstitial	8 (8.1)	2 (0.7)	
Alveolointerstitial	10 (10.1)	30 (11.1)	
Patchy radiological involvement	5 (5.0)	3 (1.1)	

ALT: alanine aminotransferase; AST: aspartate aminotransferase; CURB65: pneumonia severity score calculator 65; GGT: gamma-glutamyl transpeptidase; PSI score: pneumonia severity index score; pO_2_: O_2_ partial pressure. ^*∗*^Obtained for cases admitted at ICU.

**Table 3:** Multivariate analysis calculating the risk of being a H1N1 CAP case or control^*∗*^.

Variable	Crude OR	95% CI	Adjusted OR^*∗∗∗*^	95% CI
*Gender*				
Male	1	—	1	—
Female	1.38	0.87–2.19	1.21	0.65–2.27
*Age (years)*				
<55	1	—	1	—
55–74	0.46	0.27–0.80	1.80	0.82–3.94
≥75	0.16	0.09–0.31	0.80	0.32–2.02
*Influenza vaccine*				
No	1	—	1	—
Yes	0.35	0.2–0.61	0.80	0.29–2.17
*Pneumococcal vaccine*				
No	1	—	1	—
Yes	0.38	0.16–0.87	1.08	0.30–3.82
*CURB65* ^*∗*^				
0	1	—		
1	1.04	0.56–1.95		
2	0.36	0.19–0.68		
3	0.17	0.06–0.49		
4	n/a	n/a		
5	n/a	n/a		
*PSI score* ^*∗∗*^				
1	1	—		
2	0.89	0.40–1.97		
3	0.54	0.26–1.14		
4	0.42	0.20–0.88		
5	0.48	0.19–1.25		
*Leukocytes (cell/mm* ^*3*^)				
**≤500**	**1**	—	**1**	—
**>500**	**0.20**	**0.12–0.35**	**0.42**	**0.21–0.81**
*AST (IU/L)*				
**≤24**	**1**	—	**1**	—
**>25**	**4.74**	**2.75–8.19**	**2.60**	**1.32–5.09**
*Arthromyalgias*				
No	**1**	—	**1**	—
Yes	**4.12**	**2.4–7.10**	**2.96**	**1.40–6.26**
*Chest X-ray presentation*				
Lobar	**1**	—	**1**	—
Bilobar	**0.22**	**0.03–1.69**	**0.26**	**0.03–2.18**
Bilateral	**8.95**	**4.97–14.36**	**5.30**	**2.69–10.40**

AST: aspartate aminotransferase; CURB65: pneumonia severity score calculator 65; PSI score: pneumonia severity index score. ^*∗*^The variables which had a significant result are given in bold. ^*∗∗*^CURB65 and PSI index were excluded from multivariate analysis. All other variables were included as shown in crude analysis. ^*∗∗∗*^Adjusted OR includes gender, age, influenza vaccine, pneumococcal vaccine, leukocytes, AST, arthromyalgias, and chest X-ray presentation.

## Data Availability

The data used to support the findings of this study are available from the corresponding author upon request.
